# Association between TyG index, TG/HDL-C ratio and coronary heart disease risk in elderly postmenopausal women: a retrospective cohort study

**DOI:** 10.7717/peerj.21423

**Published:** 2026-06-12

**Authors:** Wei Wen, Qing Ye, Lixiang Zhang

**Affiliations:** Department of Cardiology, The First Affiliated Hospital of USTC, Division of Life Science and Medicine, University of Science and Technology of China, Hefei City, Anhui Province, China

**Keywords:** Triglyceride glucose index, Menopause, Coronary heart disease, Coronary angiography, Prediction

## Abstract

**Objective:**

This study aimed to explore the importance of the triglyceride-glucose (TyG) index and triglyceride (TG)/high-density lipoprotein cholesterol (HDL-C) ratio in predicting the risk of coronary heart disease (CHD) in elderly postmenopausal women.

**Methods:**

This was a retrospective cohort study conducted from March 2018 to February 2019. A total of 749 natural menopausal elderly women aged ≥60 years who were hospitalized in the Department of Cardiology, First Affiliated Hospital of the University of Science and Technology of China and underwent coronary angiography for the first time were divided into CHD group (527 patients) and non-CHD group (222 patients). The clinical data parameters used in this analysis were chosen based on literature support and clinical findings related to CHD in elderly postmenopausal women. The clinical data of the two groups were compared, and the predictive value of the TyG index and TG/HDL-C was analyzed by m ultivariable logistic regression analysis and the receiver operating characteristic (ROC) curves.

**Results:**

Multivariable logistic regression analysis showed that hemoglobin (Hb), high-sensitivity C-reactive protein (hs-CRP), TyG index, TG/HDL-C, daily sleep duration and glycosylated hemoglobin (HbA1c) were independent influencing factors of CHD in elderly postmenopausal women (*P* < 0.05). The results of Spearman correlation analysis showed that the TyG index and TG/HDL-C ratio positively correlated with the Gensini score (*r* = 0. 322 and *r* = 0.342, respectively; *P* < 0.01). The ROC curve analysis showed that the area under the ROC curve of TyG index and TG/HDL-C for predicting the risk of CHD was 0.740 (95% CI [0.703–0.777]; *P* < 0.01) and 0.771 (95% CI [0.737–0.805]; *P* < 0.01), respectively.

**Conclusion:**

This study demonstrates that the TyG index and TG/HDL-C ratio are significantly associated with CHD risk in elderly postmenopausal women, as evidenced by their acceptable predictive performance (AUC: 0.740–0.771) and independent statistical significance (*P* < 0.05). While the observed correlations with coronary artery stenosis (Gensini score: *r* = 0.322–0.342) were statistically significant but numerically weak, these markers remain clinically useful screening tools. Further validation studies are warranted to confirm their role in risk stratification.

## Introduction

Coronary heart disease (CHD) is one of the most common cardiovascular diseases (CVDs). The typical clinical manifestations of CHD include chest pain or discomfort (angina), which may radiate to the arms, neck, jaw, or back, as well as shortness of breath, fatigue, and palpitations. In severe cases, CHD may culminate in life-threatening events such as acute myocardial infarction, heart failure, or sudden cardiac death, all of which precipitate permanent and irreversible injury to the myocardium. In many countries, CHD has become the main cause of adult all-cause mortality, accounting for approximately 30.8–40% of all deaths in the world, seriously threatening and affecting the health and life of individuals (Benjamin et al., 2018). Previous studies have shown that men have an earlier age of onset of CHD than women. Furthermore, women are more predisposed to atypical symptoms—including nausea, dizziness, and unexplained fatigue—which frequently lead to delays in clinical diagnosis and intervention. As the estrogen levels in women decline after menopause, the incidence and mortality of CHD in postmenopausal women increase substantially (Khamis, Ammari & Mikhail, 2016), and the atypical symptoms of the perimenopausal syndrome are easily confused with the symptoms of CHD. These symptomatic similarities increase the likelihood of diagnostic errors, thereby elevating the risk of secondary complications and worsening overall clinical outcomes. Therefore, it is of great importance to understand the influencing factors of CHD in perimenopausal women (Liu et al., 2019a). The triglyceride-glucose (TyG) index, calculated as ln[fasting triglycerides (mg/dL) × fasting glucose (mg/dL)/2], is a reliable marker for assessing insulin resistance (IR). It is a simple, low-cost, and easy-to-apply method, and thus popularized for clinical use (Wu et al., 2021; Guo et al., 2021). The study conducted by Yan Yu et al. found that the TyG index positively correlated with the severity of coronary artery disease and closely correlated with the risk factors of CVD, which could be used as an effective tool to identify the severity of coronary artery disease (Yan & Mao, 2023). The study by Li et al. (2022) found that in patients with nonalcoholic fatty liver disease, an increase in the TyG index positively correlated with an increased risk of CHD, and the TyG index was a valuable clinical predictor of CHD risk in this high-risk group. Cheng et al. (2021) demonstrated that the TyG index is a robust indicator of CHD risk for middle-aged and elderly individuals with a normal body mass index. Their study highlighted a significant exponential correlation between elevated TyG levels and increased CHD susceptibility. Patients with CHD risk factors should closely monitor the changes in the TyG index. The ratio of triglyceride to high-density lipoprotein cholesterol (TG/HDL-C) is considered a simple surrogate index of IR (Won et al., 2018), and has been proven to be an independent risk factor for CHD, which is closely related to major adverse cardiovascular events of CHD, including CHD, death, acute myocardial infarction, and vascular remodeling of unstable angina pectoris (Prasad et al., 2019; Ingelsson et al., 2007). In addition, the study by Xiong et al. (2019) found that the TG/HDL-C ratio positively correlated with the severity of coronary artery disease in patients with CHD. Current studies predominantly concentrate on the general population; consequently, the clinical significance of the TyG index and TG/HDL-C ratio as predictors for CHD in elderly postmenopausal women remains insufficiently explored. This study aimed to evaluate the relationship between TyG index, TG/HDL-C, and CHD in elderly postmenopausal women.

The focus on elderly postmenopausal women is necessitated by the distinctive physiological and metabolic shifts associated with menopause. Notably, the postmenopausal depletion of estrogen levels profoundly alters lipid metabolism, thereby substantially elevating cardiovascular susceptibility within this demographic. While the TyG index and TG/HDL-C ratio have been widely studied in general populations, there is a paucity of research specifically examining their predictive value for CHD in this high-risk subgroup. Existing studies have primarily focused on younger or mixed populations, often overlooking the distinct cardiovascular risk profile of postmenopausal women, whose CHD presentation may differ due to hormonal influences and atypical symptoms. By concentrating on this demographic, our study addresses a critical gap in the literature, providing insights into whether these insulin resistance markers retain their predictive utility in elderly postmenopausal women, thereby guiding targeted prevention and management strategies.

## Data and methods

### Participants

The importance of this study lies in its focus on elderly postmenopausal women, a group that is particularly susceptible to CHD due to a decline in estrogen levels after menopause. By evaluating the TyG index and TG/HDL-C ratio in relation to CHD in this demographic, we aim to establish their utility as valuable biomarkers for risk assessment. These simple, cost-effective tools for assessing insulin resistance are critical, as IR is implicated in the development of CHD. Therefore, our findings carry potential implications for early diagnostic and preventive frameworks tailored to elderly postmenopausal women, with the ultimate goal of improving health outcomes and quality of life. This was a retrospective cohort study. From March 2018 to February 2019, 749 elderly female patients aged ≥60 years with natural menopause who were hospitalized in the Department of Cardiology of the First Affiliated Hospital of the University of Science and Technology of China because of precordial discomfort and underwent coronary angiography for the first time enrolled as the research participants. Based on the coronary angiography results, the patients were divided into the CHD group (527 patients) and non-CHD group (222 patients). Patients meeting any of the following criteria were excluded: (1) incomplete clinical records; (2) a history of CHD or prior coronary revascularization (*e.g.*, CABG or PCI); (3) hepatic or renal dysfunction (Wang et al., 2021); (4) concomitant cardiac conditions, including rheumatic or valvular heart disease; (5) pre-existing hematological, neurological, endocrine, or immunological disorders (Zhu et al., 2024); and (6) active acute or chronic infections.

### Data collection method

Participant data were collected retrospectively through the hospital information system and the electronic medical record system. The general clinical data including age, amenorrhea age, height and weight, presence of hypertension and diabetes, and smoking history were obtained from the admission assessment sheet in the hospital information system and general information evaluation forms in the electronic medical record system. For laboratory examination, 3–4 mL of venous blood drawn from the elbow was collected the next morning after admission in a fasting state and placed in an anticoagulant tube or dry ordinary test tube for inspection. The levels of serum creatinine, fasting plasma glucose (FPG), glycosylated hemoglobin (HbA1c), alanine aminotransferase (ALT), aspartate aminotransferase (AST), HDL-C, low-density lipoprotein cholesterol (LDL-C), TG, total cholesterol (TC), estradiol, white blood cells, lymphocytes, monocytes, cardiac troponin I (cTnI), and high-sensitivity C-reactive protein (hs-CRP) were estimated in the biochemical laboratory of the hospital. The results of the test indicators were displayed in the patient’s test results in the hospital information system on the day after the blood sample was submitted, and the results of all laboratory tests on the day of the patient’s discharge were automatically and uniformly summarized in the patient’s discharge record. The TyG index was calculated as Ln[TG (mg/dL)*FPG(mg/dL)/2]. Coronary angiography (CAG) was performed by at least two experienced cardiologists using standard percutaneous catheterization techniques. Following the administration of local anesthesia, a catheter was introduced *via* either the femoral or radial artery and advanced to the coronary ostia under fluoroscopic guidance. The Judkins technique, employing pre-shaped left and right catheters, was utilized for selective cannulation to ensure optimal opacification of the coronary anatomy. The severity of coronary artery disease (CAD) was quantified using the Gensini score. This system assigns points based on the degree of luminal stenosis (1, 2, 4, 8, 16, and 32 points for stenosis of 1–25%, 26–50%, 51–75%, 76–90%, 91–99%, and 100%, respectively) and adjusts these values by a multiplier corresponding to the anatomical significance of the affected segment (*e.g.*, ×5 for the proximal left anterior descending artery). The cumulative score represents the overall disease burden. For this study, CHD was defined as any coronary stenosis ≥50%. All angiographic data were retrieved from the hospital’s electronic information system. The study protocol was reviewed and approved by the Medical Ethics Committee of the First Affiliated Hospital of the University of Science and Technology of China (ID: 2023-RE-052). In view of the retrospective collection of research data, the hospital’s institutional review board determined that a waiver of informed consent could be applied for in this study; thus, patient informed consent was waived.

### Statistical methods

The R software (version 3.6.1) was used for data analysis. Continuous variables were expressed as mean ± standard deviation (SD) for normally distributed data and as median with interquartile range (IQR) (25th–75th percentiles) for skewed data. Categorical and ordinal variables were presented as frequencies and percentages (n (%)). For univariate analysis, group differences were compared using the independent-sample *t*-test, Mann–Whitney U test, or Pearson’s chi-square test, as appropriate based on the data type and distribution. Spearman correlation was used to analyze the correlation between two measurement data of skewed distribution. Multivariable logistic regression was chosen for risk factor analysis because it allows simultaneous adjustment for potential confounders while quantifying the independent contribution of each predictor to coronary heart disease (CHD) risk, thereby avoiding spurious associations identified in univariate analyses. Multivariable logistic regression was subsequently used to analyze the independent influencing risk factors of CHD. Receiver operating characteristic(ROC) curve was used to evaluate the predictive value of the TyG index and TG/HDL-C, and a value of *P*<0.05 was considered statistically significant.

## Results

### Comparison of general clinical data between the two groups

There was a statistically significant difference in the distribution of 21 variables (age, white blood cell count, neutrophil percentage, lymphocyte percentage, red blood cell count, Hb concentration, platelet count, hs-CRP, serum albumin concentration, cystatin, random blood glucose, TyG index, TG/HDL-C, 2-hour postprandial blood glucose, HbA1c, HDL-C, sleep duration, educational level, history of cerebrovascular disease, hypertension, and diabetes mellitus) between the CHD group and the non-CHD group (*P* < 0.05, as shown in Table 1). In addition, The Gensini score differed between the two groups (median [Q_1_, Q_3_]: 0.00 [0.00, 3.00] in the non-CHD group *vs* 40.00 [18.50, 69.00] in the CHD group; Z = −20.803; *P* < 0.001).

**Table 1:** Comparison of general clinical data between the two groups.

Indicators	Non-CHD group (*n* = 222)	CHD group (*n* = 527)	*P*
Age (years)	68.53 ± 6.33	70.27 ± 6.45	<0.001
BMI (kg/m^2^)	25.13 ± 3.42	24.79 ± 3.21	0.191
White blood cell count (cells/μL)	6.23 ± 1.67	6.70 ± 2.25	0.002
Neutrophil percentage (%)	60.90 ± 8.45	63.23 ± 9.63	0.002
Lymphocyte percentage (%)	30.06 ± 7.87	27.77 ± 8.82	<0.001
RBC (×10^12^/L)	3.96 ± 0.40	3.87 ± 0.47	0.006
Hb (g/L)	121.20 ± 11.49	117.43 ± 13.69	<0.001
PLT (×10^9^/L)	186.24 ± 51.23	201.83 ± 61.07	<0.001
hs-CRP (mg/L)	1.15 (0.46, 2.70)	4.15 (1.76, 5.00)	<0.001
ALT (IU/L)	17.00 (13.00, 22.00)	17.00 (13.00, 26.00)	0.115
AST (IU/L)	20.00 (16.00, 24.00)	20.00 (16.00, 28.00)	0.099
Serum albumin (g/L)	43.86 ± 3.91	42.74 ± 4.19	<0.001
Urea nitrogen (mmol/L)	6.88 ± 2.65	6.87 ± 2.76	0.976
Creatinine (μmol/L)	68.93 ± 38.82	72.04 ± 40.34	0.330
Uric acid (μmol/L)	318.24 ± 104.74	321.25 ± 107.86	0.726
Cystatin (mg/L)	0.98 ± 0.44	1.07 ± 0.48	0.025
Random blood glucose (mmol/L)	7.16 ± 3.06	8.12 ± 3.70	<0.001
TyG index	8.56 ± 0.46	9.10 ± 0.66	<0.001
TG/HDL-C	1.31(0.95,1.75)	2.21(1.48,4.03)	<0.001
Postprandial blood glucose (mmol/L)	7.99 ± 2.62	9.99 ± 3.98	<0.001
HbA1c (%)	5.99 ± 0.77	6.69 ± 1.36	<0.001
Total cholesterol (mmol/L)	4.45 ± 1.06	4.41 ± 1.06	0.637
HDL-C (mmol/L)	1.10 ± 0.26	1.02 ± 0.25	<0.001
LDL-C (mmol/L)	2.27 ± 0.75	2.27 ± 0.77	0.884
VLDL-C (mmol/L)	1.10 ± 0.36	1.14 ± 0.40	0.234
FT3 (pmol/L)	4.67 ± 1.15	4.59 ± 2.40	0.631
FT4 (pmol/L)	12.61 (11.62, 13.75)	12.64 (11.43, 14.02)	0.753
TSH (μIU/mL)	2.37 (1.50, 3.65)	2.43 (1.60, 3.84)	0.761
Average daily sleep duration (hrs/day)	6.55 ± 1.33	6.09 ± 1.03	<0.001
Educational level			0.001
Junior high school and below	177 (79.73%)	473 (89.75%)	
Senior high school and technical secondary school	31 (13.96%)	38 (7.21%)	
College degree or above	14 (6.31%)	16 (3.04%)	
Smoking			0.505
No	221 (99.55%)	526 (99.81%)	
Yes	1 (0.45%)	1 (0.19%)	
Drinking wine/alcohol			1.000
No	222 (100.00%)	526 (99.81%)	
Yes	0 (0.00%)	1 (0.19%)	
Complicated with cerebrovascular disease			0.009
No	186 (83.78%)	396 (75.14%)	
Yes	36 (16.22%)	131 (24.86%)	
Complicated with hypertension			0.034
No	81 (36.49%)	151 (28.65%)	
Yes	141 (63.51%)	376 (71.35%)	
Diabetes mellitus			<0.001
No	184 (82.88%)	337 (63.95%)	
Yes	38 (17.12%)	190 (36.05%)	

**Notes.**

Abbreviations BMI body mass index WBC white blood cell count RBC red blood cell count Hb hemoglobin PLT platelet count hs-CRP high-sensitivity C-reactive protein ALT alanine aminotransferase AST aspartate aminotransferase TyG index triglyceride-glucose index TG/HDL-C triglycerides to high-density lipoprotein cholesterol ratio HbA1c glycated hemoglobin HDL-C high-density lipoprotein cholesterol LDL-C low-density lipoprotein cholesterol VLDL-C very low-density lipoprotein cholesterol FT3 free triiodothyronine FT4 free thyroxine TSH thyroid-stimulating hormone.

### Result of multivariable logistic regression analysis

The variables with *P* < 0.05 screened in Table 1 were used as independent variables, and the incidence of CHD in elderly menopausal women was used as a dependent variable to construct a binary Logistic regression model. The inclusion of variables (*e.g.*, sleep duration, Hb levels) in the logistic regression model was exploratory, as they were initially selected based on univariate screening (*P* < 0.05) rather than a predefined hypothesis. Backward method was used for variable screening based on a significance level of *P* < 0.05. Among the 21 indicators selected in Table 1, continuity variables were directly brought into the regression model, and the remaining categorical variables were classified by one-hot encode processing and then brought into the regression model. The analysis results showed that Hb and daily sleep duration were independent protective factors of CHD in elderly postmenopausal women (odds ratio (OR) < 1 and *P* < 0.05), and hs-CRP, TyG index, TG/HDL-C and HbA1c were independent risk factors of CHD in elderly postmenopausal women (OR > 1 and *P* < 0.05), as shown in Table 2.

**Table 2:** Multivariable logistic regression analysis of the risk of coronary heart disease in elderly postmenopausal women.

Indicators	β	SE	*Z*	*P*	OR	95% of OR
Hb	−0.022	0.009	−2.403	0.016	0.979	0.962∼0.996
Hs-CRP	0.410	0.059	7.004	<0.001	1.507	1.343∼1.690
TyG index	1.532	0.246	6.221	<0.001	4.628	2.856∼7.499
TG/HDL-C	0.710	0.111	6.379	<0.001	2.034	1.635∼2.530
Average daily sleep duration	−0.341	0.093	−3.671	<0.001	0.711	0.593∼0.853
HbA1c	0.376	0.129	2.926	0.003	1.457	1.132∼1.875

**Notes.**

Abbreviations Hb hemoglobin Hs-CRP high-sensitivity c-reactive protein TyG index triglyceride-glucose index TG/HDL-C triglyceride to high-density lipoprotein cholesterol ratio HbA1c hemoglobin a1c OR odds ratio SE standard error β beta coefficient

### Spearman correlation analysis of TyG index, TG/HDL-C, and Gensini score

**Figure 1:**
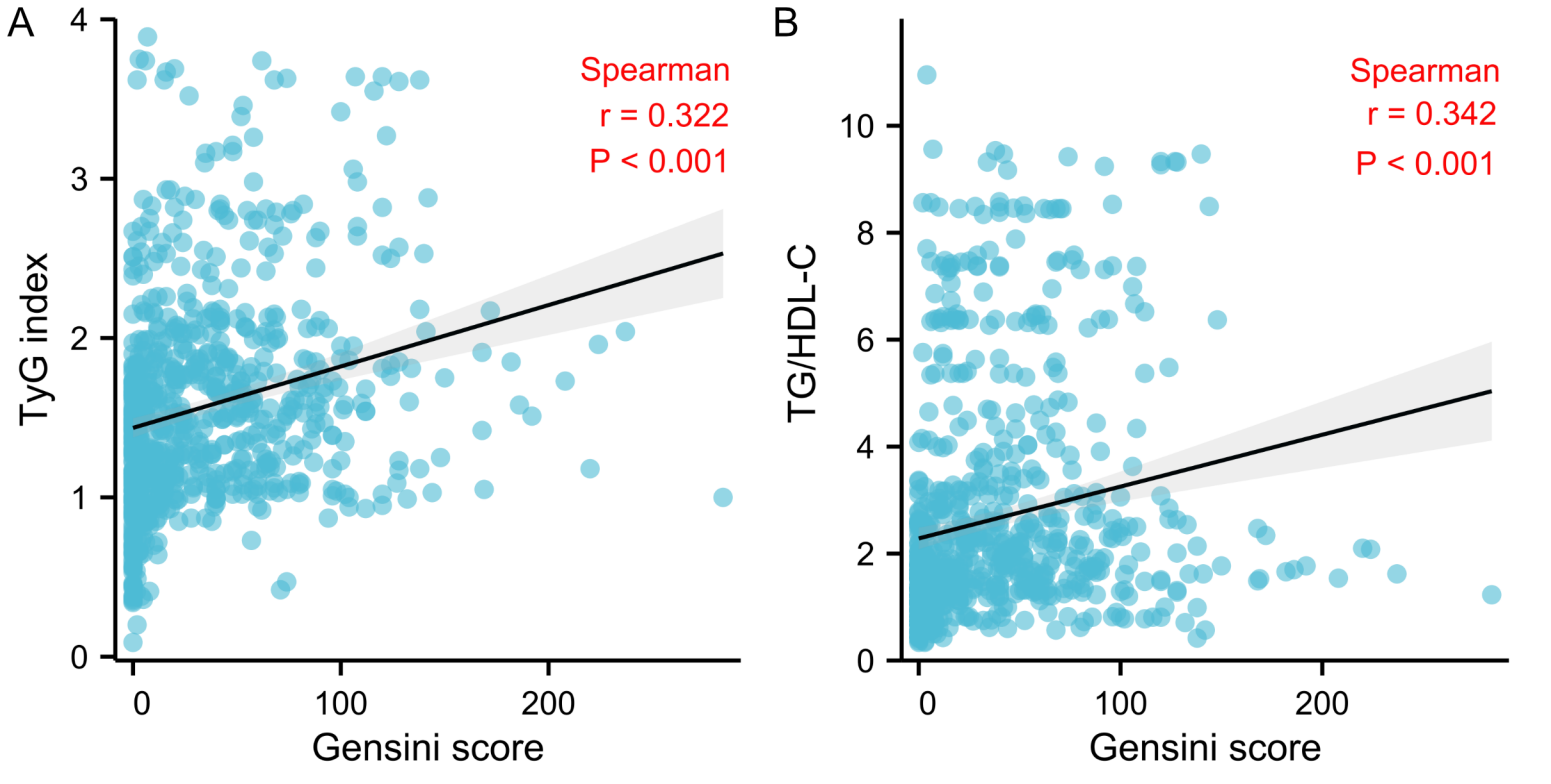
Scatterplot of correlation between TyG index, TG/HDL-C and Gensini score. (A) Scatter plot showing the positive correlation between the TyG index and the Gensini score. Each point represents an individual patient’s data. The black line indicates the trend line, and the shaded area represents the 95% confidence interval. The Spearman correlation coefficient (*r*) is 0.322, and the *P*-value is less than 0.001, indicating a statistically significant positive correlation. B: Scatter plot depicting the positive correlation between the TG/HDL-C ratio and the Gensini score. Similar to (A), each point corresponds to an individual patient’s data. The trend line is shown in black, with the 95% confidence interval indicated by the shaded area. The Spearman correlation coefficient (*r*) is 0.342, and the *P*-value is less than 0.001, suggesting a statistically significant positive correlation.

The results of Spearman correlation analysis showed that the TyG index and TG/HDL-C ratio positively correlated with the Gensini score (*r* = 0.322 and 0.342, respectively *P* < 0.01), as shown in Fig. 1.

### ROC curve analysis of the predictive value of TyG index and TG/HDL-C

**Figure 2:**
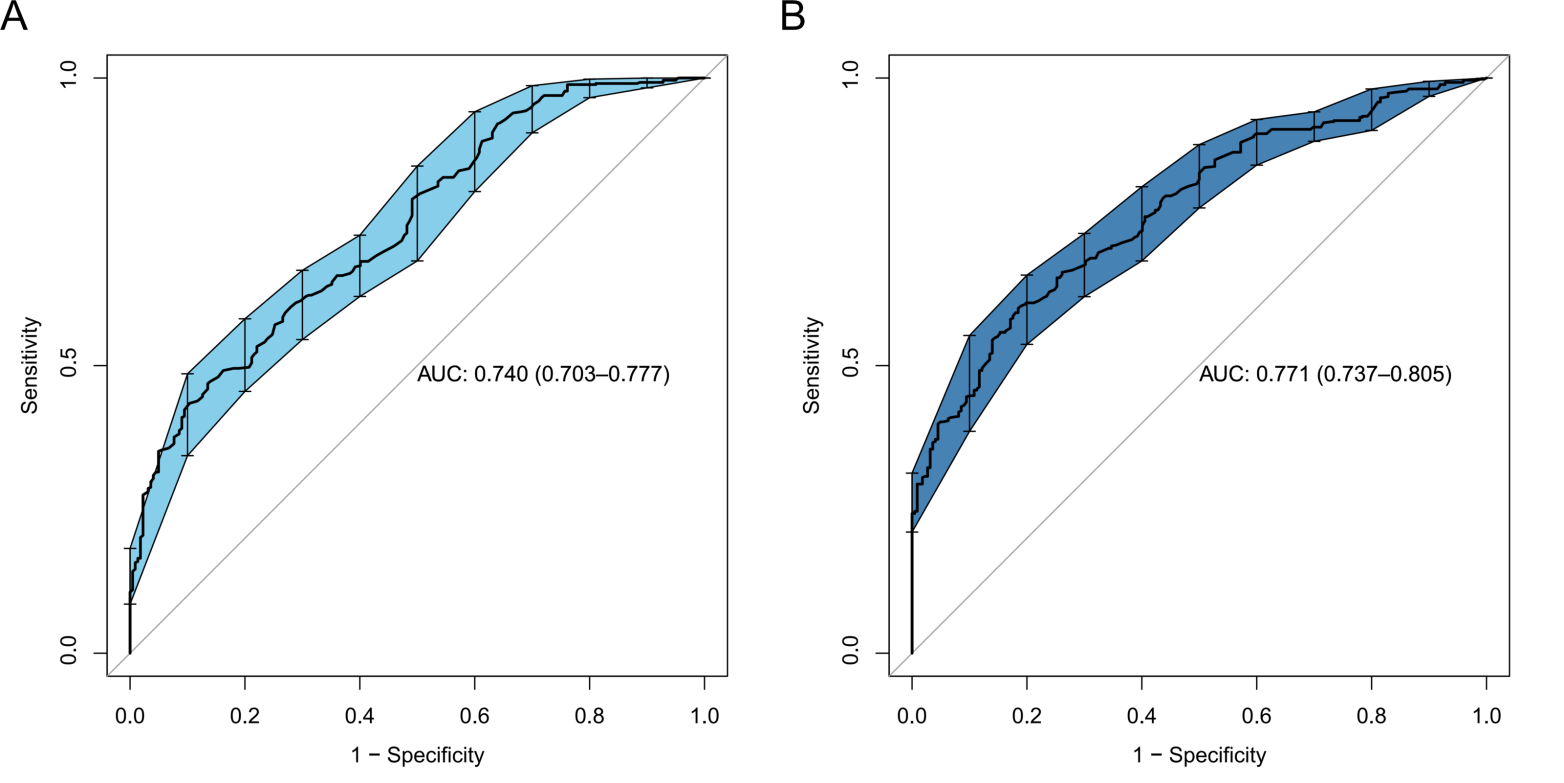
ROC curve analysis of TyG index, TG/HDL-C. (A) ROC curve for the TyG Index. The trade-off between sensitivity and 1-specificity for the TyG Index in predicting coronary artery disease. The area under the curve (AUC) is 0.740 with a 95% confidence interval of 0.703 to 0.777, indicating a moderate level of discriminative ability. The gray line represents the reference line, which is the ROC curve for a test with no diagnostic ability (AUC = 0.5). The closer the curve follows the left-hand border and then the top border of the ROC space, the more accurate the test. B: ROC curve for the TG/HDL-C ratio. This curve shows the performance of the TG/HDL-C ratio in predicting coronary artery disease, with an AUC of 0.771 and a 95% confidence interval of 0.737 to 0.805, suggesting a slightly better discriminative ability compared to the TyG Index. The shaded areas around the curves in both panels represent the 95% confidence intervals for the AUC estimates. The gray line in each panel also represents the reference line, indicating no diagnostic ability. The proximity of each curve to the left and top borders of the ROC space is indicative of the test’s accuracy.

The area under the curve (AUC) of the TyG index for assessing the risk of CHD in elderly menopausal women was 0.740 (95% CI [0.703∼0.777]; *P* < 0.01), and the AUC of TG/HDL-C was 0.771 (95% CI [0.737∼0.805]; *P* < 0.01). As shown in Fig. 2. The optimal cutoff values of the TyG index and TG/HDL-C for predicting the risk of CHD were 9.045 and 1.855, respectively (Table 3).

### Discussion

Research indicates that the incidence of coronary heart disease in postmenopausal women aged 65 and above is 43.3% (45 out of 104 cases) (Li, Zhao & Tu, 2004). Further studies by Zhang et al. (2009) revealed that the rate of positive coronary angiography in postmenopausal women with chest pain can reach up to 79.3%, compared to 35.0% in reproductive-aged women. Zhen (2013) research found that among 215 patients with chest pain, the positive rate of coronary angiography was 67.7% (21/31) for females, but increased to 75.0% in the elderly female group. De Vault et al. (2004) study revealed that postmenopausal women (average age 59 years) had a positive rate of coronary angiography at 67.14% (47/70). Wang Wang et al. (2008) research indicated that elderly women (≥52 years) with typical chest pain and positive exercise tolerance test (ETT), the positive rate of coronary angiography could reach 86%.

In the present study, the prevalence of positive coronary angiography among elderly postmenopausal women undergoing their initial evaluation for precordial symptoms was 70.4% (527/749). This finding is congruent with the previously cited literature. The discrepancy in the positive coronary angiography rates among postmenopausal women with chest pain across different studies may be related to variations in sample size, age criteria, and study duration. Studies have shown that CVD is the primary cause of high mortality in postmenopausal elderly women (Cushman et al., 2021). Compared with men, female patients with CHD symptoms are less noticeable and evident, coupled with the lower social and family status and lack of awareness of the disease in elderly women. In clinical work, the rate of missed diagnosis of CHD in female patients is high, and the rate of treatment is low. Therefore, increased focus and consideration should be given to prevent CVDs in elderly women (Liao et al., 2021).

The TyG index is a novel marker with high sensitivity and specificity in identifying metabolic syndrome, and its reliability as a surrogate marker of IR has been demonstrated in previous studies (Angoorani et al., 2018). IR is defined as a pathological state characterized by the diminished responsiveness of target organs to insulin action. This condition primarily manifests as impaired glucose uptake and utilization within insulin-sensitive tissues—specifically the liver, adipose tissue, and skeletal muscle. These metabolic aberrations result in systemic glucose and lipid dysregulation, which subsequently drives the pathogenesis and progression of various metabolic disorders. IR directly causes atherosclerosis and is associated not only with an increased risk of CVD but also with a significantly higher risk of cardiovascular outcomes (Rutter et al., 2005). Recent studies have found that the TyG index is directly related to the risk of CVD (Lee et al., 2016). Other studies have found that the TyG index is significantly associated with coronary artery calcification (Kim et al., 2017). Research by Liu et al. (2023) found that a higher baseline TyG index, as a marker of IR, is linked to an increased risk of cardiovascular disease (CVD) in postmenopausal women. The TyG index could serve as a straightforward and practical tool for early identification of high-risk individuals in this population. Congruently, research conducted by Liu et al. (2022) demonstrated that the TyG index possesses predictive utility for CAD risk within postmenopausal populations. The findings of this study align with those of the two studies mentioned above. However, this study specifically focused on older postmenopausal women aged 60 and above, whereas the previous studies included women under 50 years of age. The results of this study showed that the TyG index was an independent risk factor for the occurrence of CHD in elderly menopausal women (*P* < 0.05) and was positively correlated with the degree of coronary artery stenosis (*r* = 0.322, *P* < 0.05). The possible mechanism is that the increase in the TyG index reflected a more severe IR, which further promoted the incidence of CVDs by reducing the production of nitric oxide, inducing vascular endothelial dysfunction, and promoting the formation of atherosclerotic plaques (Hill et al., 2021). Additionally, the TyG index, as a marker of IR, contributes to coronary narrowing by modulating endothelial impairment and systemic inflammatory responses. Diminished nitric oxide bioavailability, a hallmark of IR, leads to dysfunctional vasoconstriction and increased arterial rigidity. These processes are further compounded by chronic hyperglycemia and hypertriglyceridemia, which promote oxidative stress and lipid sequestration, thereby driving the advancement of coronary atherosclerosis. Elevated TyG levels also enhance macrophage activation and foam cell formation, accelerating plaque instability and luminal narrowing in coronary arteries.

**Table 3:** Effect analysis of TyG index and TG/HDL-C in assessing the risk of coronary heart disease.

Indicators	AUC	Optimum cut-off value	Specificity	Sensitivity	Prediction accuracy	Positive predictive value	Negative predictive value
TyG index	0.740	9.045	0.865	0.469	0.586	0.892	0.407
TG/HDL-C	0.771	1.855	0.815	0.600	0.664	0.885	0.462

**Notes.**

Abbreviations TyG index triglyceride-glucose index TG/HDL-C triglyceride to high-density lipoprotein cholesterol ratio AUC area under the curve

Gaziano et al. (1997) first reported the correlation between TG/HDL-C ratio and acute myocardial infarction and were the first to propose that the ratio could be used to predict the risk of myocardial infarction and other cardiovascular events. Since then, many clinical studies (Hadaegh et al., 2009; Paramsothy et al., 2010) have found that compared with normal population, patients with elevated TG/HDL-C ratio have significantly increased risk of CHD, and the incidence and mortality of cardiovascular events are also significantly higher than those in normal population, suggesting that the TG/HDL-C ratio has prognostic value in early diagnosis and assessment of CHD. While the TG/HDL-C ratio is a marker in broader populations, its specific predictive value and relationship with coronary artery disease severity in elderly postmenopausal women have remained unexplored. The results of this study showed that TG/HDL-C was an independent risk factor for the occurrence of CHD in elderly menopausal women (*P* < 0.05) and positively correlated with the degree of coronary artery stenosis (*r* = 0.342, *P* < 0.05). The possible underlying mechanism as suggested by the previous studies was that both high TG level and low HDL-C level were important markers of CVD (Han et al., 2016; Shapiro & Fazio, 2016). High TG levels can damage vascular endothelium, lead to endothelial dysfunction, promote coagulation, and activate inflammatory reactions *in vivo*, eventually leading to atherosclerosis (Liu et al., 2019b). In addition to its role in mediating reverse cholesterol transport, HDL-C plays a protective role owing to its anti-inflammatory and antioxidant properties (Gao et al., 2019). As a composite metric, the TG/HDL-C ratio reflects both pro-atherogenic TG levels and anti-atherogenic HDL-C concentrations through its inherent positive and negative correlations, respectively. This relationship facilitates a more nuanced assessment of the overall atherogenic burden in elderly postmenopausal women. Additionally, an elevated TG/HDL-C ratio reflects atherogenic dyslipidemia, characterized by an excess of triglyceride-rich lipoproteins and dysfunctional HDL, which promotes coronary stenosis. High triglycerides increase small, dense LDL particles that readily infiltrate the arterial wall, while low HDL impairs reverse cholesterol transport, allowing plaque buildup. Furthermore, this lipid profile triggers pro-inflammatory cytokine release and endothelial damage, exacerbating atherosclerosis and coronary artery narrowing.

The TyG index and TG/HDL-C ratio are valuable biomarkers for assessing CHD risk, particularly in elderly postmenopausal women. The AUC values of 0.740 (TyG index) and 0.771 (TG/HDL-C) demonstrate good predictive accuracy, indicating their clinical utility in identifying individuals at higher risk. The optimal cutoffs—9.045 for the TyG index and 1.855 for TG/HDL-C—were derived from this population and reflect thresholds beyond which CHD risk significantly increases. These cutoffs are clinically relevant because they help stratify patients for early intervention, such as lifestyle modifications or pharmacological therapy, to mitigate cardiovascular risk. Although the present diagnostic thresholds were established specifically within an elderly postmenopausal cohort, their external validity across diverse populations warrants further investigation. Potential metabolic and hormonal variations inherent to different demographic contexts may modulate the predictive accuracy and clinical utility of these biomarkers. Nonetheless, these findings underscore the importance of insulin resistance (TyG index) and atherogenic dyslipidemia (TG/HDL-C) as key contributors to CHD risk in this demographic.

## Conclusion

The TyG index and TG/HDL-C ratio are significantly associated with CHD risk in elderly postmenopausal women, as evidenced by their acceptable predictive performance (AUC: 0.740–0.771) and independent statistical significance (*P* < 0.05). While the observed correlations with coronary artery stenosis (Gensini score: *r* = 0.322–0.342) were statistically significant, but the effect sizes were numerically weak, these markers remain clinically useful screening tools. These findings highlight the translational value of the TyG index and TG/HDL-C ratio for clinical practice, suggesting that they can be used as reliable markers to assess CHD risk in this specific patient population, potentially improving early detection and management strategies. However, this is a single-center study with a small sample size, which may introduce selection bias and limit the generalizability of the findings. Additionally, potential confounding variables such as socioeconomic status, dietary habits were not accounted for in the analysis. These factors could influence both the TyG index and TG/HDL-C ratio, as well as the risk of CHD, potentially biasing the observed associations. Furthermore, the retrospective nature of data collection may introduce recall bias or misclassification of exposures and outcomes, as it relies on previously recorded information that may be incomplete or inaccurate. Future large-scale, multicenter studies incorporating comprehensive adjustments for these confounders are needed to further validate the predictive value of the TyG index and TG/HDL-C ratio in elderly postmenopausal women with CHD.

## Supplemental Information

10.7717/peerj.21423/supp-1 Supplemental Information 1 Raw data

## Abbreviations

CHD: Coronary heart disease
HDLC: High-density lipoprotein cholesterol
TyG: Triglyceride-glucose
IR: Insulin resistance
FPG: Fasting plasma glucose
TC: Total cholesterol
HbA1c: Glycosylated hemoglobin
TG: Triglycerides
ALT: Alanine aminotransferase
LDL-C: Low-density lipoprotein cholesterol
AST: Aspartate aminotransferase
HDL-C: High-density lipoprotein cholesterol
cTnI: Cardiac troponin I
SE: Indicates standard error
hs-CRP: high-sensitivity C-reactive protein
β: Regression coefficients
OR: Odds ratio
95% CI: 95% confidence interval
ROC: Operating characteristic curve
AUC: Area under the ROC curve

## Additional Information and Declarations

### Data Availability

The following information was supplied regarding data availability: The raw data is available in the Supplemental File.
